# Near and Far-Field Properties of Nanoprisms with Rounded Edges

**DOI:** 10.1007/s11468-014-9671-x

**Published:** 2014-03-07

**Authors:** Bartłomiej Grześkiewicz, Krzysztof Ptaszyński, Michał Kotkowiak

**Affiliations:** Faculty of Technical Physics, Poznan University of Technology, Nieszawska 13a, 60-965 Poznań, Poland

**Keywords:** Optical property, Triangular nanoprism, Bowtie, Rounded edges, Finite Integration Technique

## Abstract

Photonic devices can be developed, and their working principle can be understood only by considering the phenomena taking place at the nanoscale level. Optical properties of plasmonic structures depend on their geometric parameters and are sensitive to them. Recently, many advanced methods for the preparation of nanostructures have been proposed; however still, the geometric parameters are inaccurate. Numerical simulations provide a powerful tool for the analysis of plasmonic nanostructures. To the best of our knowledge, there are not many papers on near-field and far-field properties of single nanoprism and nanoprism dimer, the so-called bowtie, with rounded edges. For this purpose, Finite Integration Technique implemented to the CST Microwave Studio was used. Besides the edge rounding, an additional modification of the resonance modes was investigated, achieved by placement of a spherical nanoparticle in the gap between the prisms. Results of numerical simulations indicate that the radius of the curvature edges strongly affects the plasmon peak localization, and this effect cannot be neglected in plasmonic device design. Increase in the radius of edge curvature causes main extinction cross-section peak blueshift in all cases analyzed. Moreover, our calculations imply that the nanoparticle in the gap between prisms strongly influences the dependence of spectral properties on the radius curvature.

## Introduction

Noble metal nanostructures can strongly enhance an electric field upon incident light illumination due to the phenomenon called localized surface plasmon resonance. Collective oscillations of quasi-free electrons on the surface of metallic structures and their interactions with molecules lead to the observed surface enhancement of Raman and fluorescence signals [1, 2]. A lot of structures with different shapes have been hitherto theoretically and experimentally investigated, while plasmonic properties strongly depend on the morphology of the nanostructures [3–8]. One of the up-to-date and the most interesting structures is nanoprisms [4, 5, 8]. Nowadays, many advanced methods are used for nanostructure fabrication, including chemical synthesis of nanoparticles and the bottom-up approach [9–12]. Nevertheless, it is not possible to obtain perfect geometric parameters, e.g., sharp edges of nanoprisms or well-defined distance between nanostructures [13]. Theoretical simulations can predict how morphological changes of nanostructures influence their near- and far-field properties. The most common methods are the following: Finite difference time domain method (FDTD), discrete dipole approximation (DDA), and Finite Integration Technique (FIT) [5, 7, 14].

The strong local field enhancement in a single nanoprism seems to be suitable for various applications. Intensity of electric field can be increased using nanoprism dimers with perfectly sharpened edges [13]. It has been noted that enhancement of near-field optical intensity at the corner with nonzero radius can be correctly predicted, but the influence of the curvature of the corners of the mono and dimer nanoprisms on far-field properties has only been little studied [13, 15, 16].

The influence of rounded edges on the optical properties has been experimentally detected or theoretically simulated for different structures. Qian et al. [17] have observed the blueshift of extinction peaks for nanoboxes with modified inner and outer edge rounding. McMahon et al. [18], for silver nanocubes, found a correlation between the changes in geometrical dimensions, i.e., corner rounding, and their optical response. Raziman and Martin [19] have considered the rounding of nanorod antennae and observed significant changes in the scattering far-field properties. Goldys et al. [20] have pointed out that only by taking into account real geometry, it is possible to produce results similar to the experimental ones.

The process of fabrication of metal nano-objects always introduces some rounding effect on the structures. This effect must be incorporated into the simulation process in optical response prediction [13, 18, 19]. As follows from the points mentioned above, the shape of the fabricated structure is always different from the ideal one. If the real geometry of nanostructures is taken into account during numerical calculations, a good agreement between the experimental and theoretical predictions could be obtained. The relationship between the optical response, structure, and dielectric environment of nanostructures is important to effectively design the devices employing their plasmonic properties [18].

In this paper, the extinction spectra and electric near-field enhancement/distribution of single and dimer Au nanoprism with rounded edges were investigated by the FIT method; this calculation method is implemented into CST Microwave Studio software (CST MWS) (www.cst.com). What is more, the modification of resonance modes of nanoprism dimer by a single nanoparticle (NP) in the gap between them was also examined. In order to modify plasmonic modes, NP made of different materials was used, including metallic and insulating phases.

## Methods

FIT [21, 22] provides discrete reformulation of the integral form of Maxwell’s equations. The volume restricted by boundary conditions is divided into a set of polyhedral unit cells called mesh. Then the linear integral can be written as the algebraic sum of discrete products along the border of the mesh faces, and the surface integral as the flux through the mesh face. The equations of all faces can be collected in matrix form. For example, Faraday’s law can be expressed as (1):

1 $$ {\displaystyle \underset{\delta A}{\oint }}\overrightarrow{E}\cdot d\overrightarrow{s}=-{\displaystyle \underset{A}{\iint }}\frac{\partial \overrightarrow{B}}{\partial t}\cdot d\overrightarrow{A} $$

where $\overrightarrow{E}$ and $\overrightarrow{B}$ are the electric and magnetic field, respectively, and $d\overrightarrow{s}$ and $d\overrightarrow{A}$ are the infinitesimal vector element of the contour *δA* and an infinitesimal vector element of surface *A*, respectively.

Faraday’s law (1) can be discretized to yield its counterpart written in the matrix form given by (2):

2 $$ \widehat{\boldsymbol{C}}\boldsymbol{e}=-\frac{d\boldsymbol{b}}{ dt} $$

where ***e*** and ***b*** are the vectors of electric voltages on the mesh edges and magnetic fluxes on the mesh faces, and $\widehat{\boldsymbol{C}}$ is the matrix representing the curl operator, containing only −1, 0, and 1 coefficients.

All simulations presented in this paper use the CST MWS’s frequency domain solver with a tetrahedral mesh, the method used before by Dyck et al. [23]. Tetrahedral grid provides flexibility in approximating arbitrary (i.e., rounded) geometries, while hexahedral mesh gives poor approximation unless a very fine mesh is used [24].

In this paper, the FIT method is used to calculate the optical extinction, radar, and absorption cross sections (i.e., ECS, RCS, ACS, respectively), and the electromagnetic field enhancement distribution ($\left|\overrightarrow{E}\right|/\left|\overrightarrow{E_0}\right|$, where $\left|\overrightarrow{E_0}\right|$ is the magnitude of the incident field, and $\left|\overrightarrow{E}\right|$ is the magnitude of the local electric field).

**Fig. 1:**
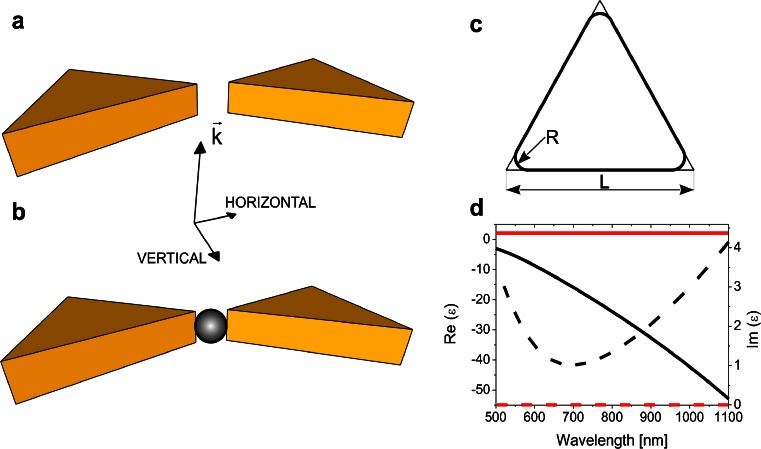
Geometry of Au bowtie: without (**a**) and with (**b**) nanoparticle structure in the gap, single rounded triangular nanoprism (**c**), and dispersion behavior **(d**) of Au (*black*) and SiO_2_ (*red lines*) (real part—*solid*, imaginary part—*dashed lines*). Color figure online

Figure 1a, b presents the model of an Au bowtie structure, used in the numerical simulations, before and after placement of 30-nm dielectric (SiO_2_) or metallic (Au) NP in the gap. The gap size is fixed at 30 nm. Figure 1c illustrates the structure of a single-rounded Au triangular nanoprism being a part of a bowtie. In all calculations, the height of triangular nanoprism and the size of the prism’s side are fixed at 30 and 150 nm (L, Fig. 1c), while the radius of curvature of the corners (*R*) is changed in the range between 0 and 10 nm (in detail 0, 0.625, 1.25, 1.875, 2.5, 3.75, 5, 6.25, 7.5, 8.75, and 10 nm). In order to ensure the comparability of the results for different radii of curvature, the size of the elementary cell was kept constant.

The bulk dielectric permittivities ε used in the calculations are obtained from the experiment [25]. Dispersion relations of the materials used are presented in Fig. 1d. In the range of our interest, dielectric permittivity of SiO_2_ has approximately constant real part Re(ε) and imaginary part Im(ε) close to zero. The real part of dielectric permittivity of Au has a negative value which monotonically decreases as wavelength increases, while the imaginary part is positive and has a minimum at ∼700 nm.

## Results and Discussion

**Fig. 2:**
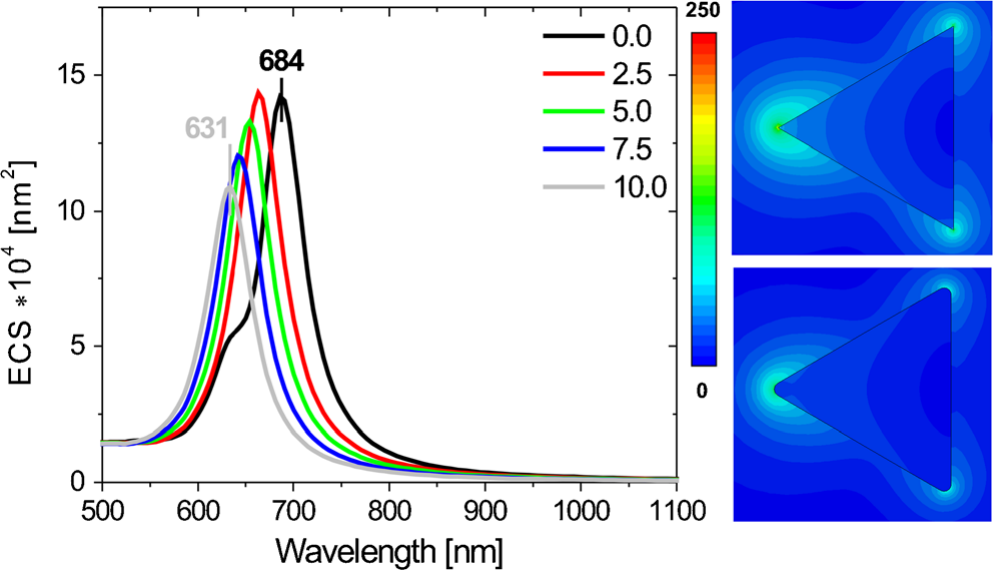
Example of extinction cross-section spectra for different edge roundings of a single nanoprism for longitudinal polarization of incident light and electric field enhancement for *R* = 0 nm. *R* = 5 nm is calculated at the maximum of corresponding extinction cross-section spectrum

**Fig. 3:**
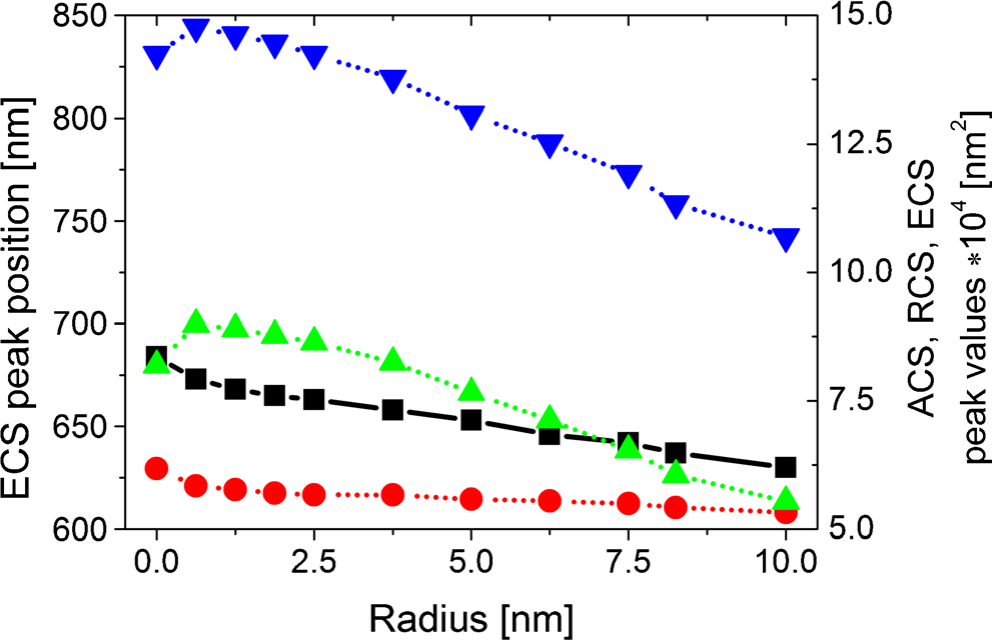
Dependencies of ECS peak position and ACS, RCS, and ECS peak values versus the edge rounding of single nanoprism for longitudinal polarization of incident light (*black line* and *symbol*: ECS peak position, whereas *red*, *green*, and *blue* correspond respectively to ACS, RCS, and ECS peak values)

Numerical studies started with an investigation of the optical properties of a single Ag triangular nanoprism with sharp and rounded edges. Earlier experimental results obtained for single nanoprism suggested that the shape of the corner region is the key factor to obtain a large field intensity enhancement and to shape the local field distribution [13, 16]. A scheme of this type of nanostructure with characteristic geometric dimensions is presented in Fig. 1c. The edge rounding *R* is varied from 0 to 10 nm. We assume that the nanoprisms are placed in a vacuum and are always illuminated in the same way from the top by the incident light with horizontal (longitudinal) or vertical (transversal) polarization (see Fig. 1a). Optical extinction cross section (ECS) as a function of wavelength for the nanoprisms with sharp and rounding edges is shown in Fig. 2. Extinction peaks correspond to the in-plane dipole resonance, as evidenced by the near-field analysis (see Fig. 2). It is distinctly observed in Fig. 3 that the rounding of edges causes a nearly linear blueshift of ECS maximum. We suppose that the change in size partially accounts for the spectral shift. Size dependence of spectral properties of metallic nanoparticles is a well-known phenomena, and its explanation is beyond the scope of this article. A few analytical models of optical properties have been proposed for some geometries, for example, the Mie theory explains the size dependence of spectral properties of spherical nanoparticles [26], and the optical antenna theory can be successfully applied to describe the optical properties of nanorods [27]. Qualitatively, this shift can be explained by decreased charge separation causing an increase in the restoring force, resulting in higher plasmon resonance frequency [8, 17] and phase retardation connected with time delay of reaction of charges on the one side of the particle to the change in charge distribution on the other side [28]. A change in the position of ECS peak, between the extreme radii considered, Δλ = λ(*R* → 10)–λ(*R* → 0) is noticeable and reaches 53 nm. The L to *R* ratio is changed from 0 to 6.6 %, and because of this, *R* has influence on the side length changes. The rounding of the edges makes the nanoprism smaller in size, which would additionally translate into a spectral shift of the resonances. Shuford et al. [29] have presented theoretical studies on the optical properties of gold triangular nanoprisms and determined the effect of structural modification on the extinction spectrum, by means of DDA calculations. They have found well-defined trends in the particle extinction to depend on the triangular edge length and height and the prism thickness. The wavelength corresponding to the peak extinction maximum increases linearly with the edge length for particles of the same height. What is more, with increasing prism height, the slope of linear dependencies between peak position and prism length decreases. If we assume that the length of the prism is 150 nm and its height is 20 nm, a 10 % change in the prism length causes a blueshift of the main extinction peak by 25 nm [29]. We suppose that the change in the length cannot account for the whole spectral shift, and additional reasons for the blueshift must be considered. Raziman and Martin [19] have connected the blue shift with the spread out of charge distribution. The reason for blueshift is the reduction in the lightning rod effect, manifested as a decrease in the highest concentration and enhancement of electromagnetic field (charge accumulation) at the sharp tips [8, 17, 30, 31]. Other authors [13, 18] have observed this effect for single nanoprism and hollow metal boxes as with increasing radius of their curvature; the charge distribution becomes less localized when compared to *R* = 0, because the Coulomb interactions between charges at the corner are reduced.

As expected, the nanoprisms with rounded edges have smaller ECS peak than the prisms with sharp edges. Absorption cross section (ACS), radar cross section (RCS), and ECS peak values decrease with increasing *R*, and also RCS to ACS ratio decrease. We suppose that it is a result of the reduction in geometrical size of nanoprism, as the same effect is predicted by the analytical theory of light scattering by spherical particles [26], and confirmed by experimental results for triangular prisms [8].

The extinction spectrum curve for the sharp edge nanoprism differs from the other curves in Fig. 2. On the left side of the main peak of ECS, a shoulder at 630 nm is clearly observed. We suppose that it is a result of the splitting of dipole resonance caused by the presence of sharp edges, as the same effect has been reported in [30, 32]. In the case of single prisms, the local maximum enhancement of electric field generated near the surfaces of nanoprism with sharp (*R* = 0) and rounding edges (*R* = 2.5 and 5 nm) reaches values of 285, 167, and 103 nm, respectively. The local surface plasmon resonance and the scattering (RCS) spectra depend on the rounding of the prism corners. The changes in local field enhancement are related to the dependence of the distribution of surface charges induced by plasmon excitation at the corner [13]. Despite the general trend, it is easy to notice abnormality. The nanoprism with *R* ∈ < 0.625, 2.5 > nm is characterized by stronger scattering (RCS) of incident light, relative to that of *R* = 0 (Fig. 3). In our opinion, this is a consequence of the resonance splitting effect [32]. It is known, that the interaction of different modes may lead to changes in the RCS spectral line shape due to Fano resonance caused by phenomena of destructive or constructive interference [33]. A similar abnormality has been indicated in the article of Raziman and Martin [19] concerning silver nanocubes, but it has not been discussed by the authors. In order to confirm the validity of our results, we have performed convergence test for *R* of 0 and 2.5 nm, which proved the convergence of our result within the error of calculations.

**Fig. 4:**
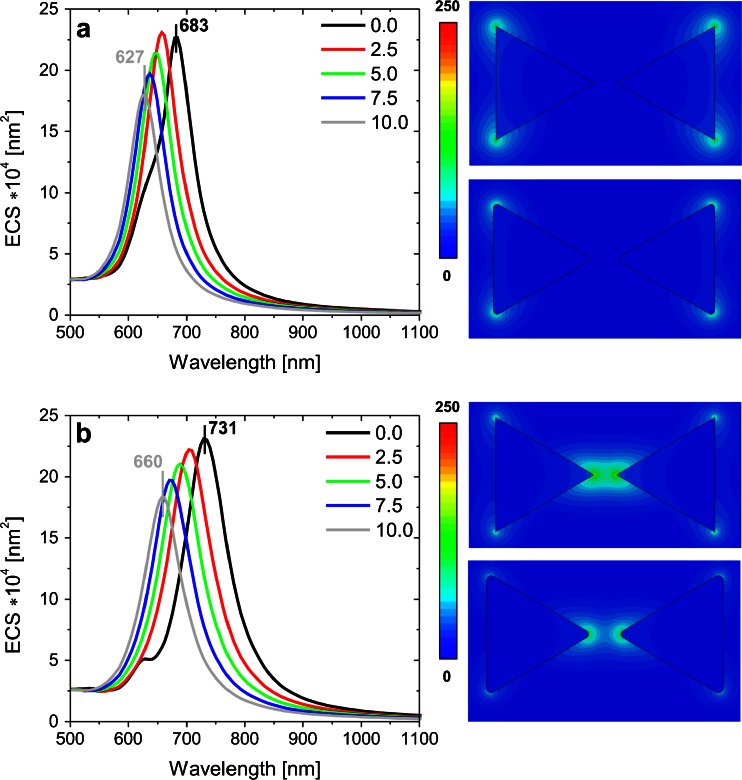
Extinction cross-section spectra of a bowtie for transversal (**a)** and longitudinal (**b)** light polarizations and electric field enhancement for *R* = 0 nm. *R* = 5 nm is calculated at the maximum of corresponding extinction cross-section spectrum

In the next step, the influence of edge rounding (*R*) on optical efficiencies of dimer nanoprisms was studied. Dimer nanoprisms, also called bowtie structures, are well known from literature [5] and are schematically presented in Fig. 1b. The optical properties of bowtie structures are strongly dependent on the direction of incident light polarization, and therefore, we considered transversal and longitudinal polarizations (see Fig. 1). Figure 4 shows the tunable ECS spectra for bowtie as a function of edge rounding. The shape of the spectral curves, the position of ECS maximum, and local electric field enhancement in a bowtie for transversal polarization (Fig. 4a) are similar to those obtained for a single nanoprism (Fig. 2). In the analyzed case (Fig 4a), the influence of resonance splitting for *R* = 0 is also clearly observed. On the basis of the above insights and literature data [5], we conclude that in transversal polarization, only the weak dipole-dipole coupling effect is observed. In contrast, for longitudinal polarization, the strong dipole-dipole coupling effect in bowtie is noticeable [5]. The consequences of this effect are a stronger electric field in the midgap, much broader and more intense ECS peaks for the longitudinal than for transversal polarization. Larger blueshift as a function of edge rounding in Fig. 4b in comparison with Figs. 2 and 4a is caused not only by decreasing the size (scaling effect) and changing the shapes (lightning rod effect) of nanoprisms but mainly by increasing the midgap distance. As follows from theoretical considerations, based on the dipole-dipole interaction model [30], which for significant midgap distance gives results consistent with the more sophisticated hybridization model [34] and has been experimentally confirmed, the midgap distance increase upon increasing edge rounding causes a weaker dipole-dipole coupling effect in the Au bowtie, and consequently a shift in the resonance peak toward higher energies. In the hybridization model, the shift is explained by the bonding combination of nanoparticle plasmons [35].

**Fig. 5:**
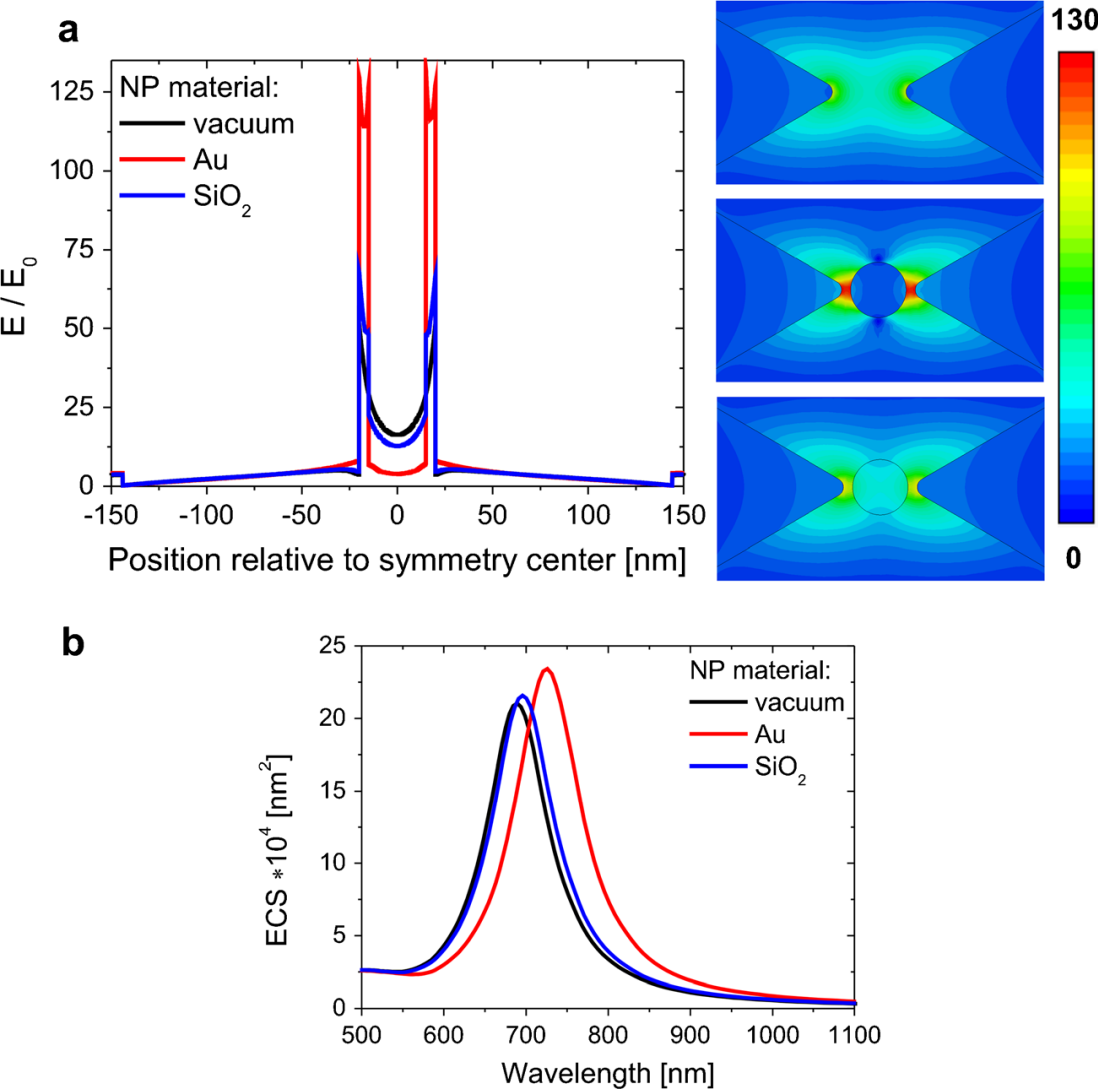
Electric field enhancement along the longitudinal symmetry axis of bowtie and the corresponding electric field enhancement (**a**). Example of extinction cross-section spectra (**b**) for different NP materials (vacuum, Au, SiO_2_) (*R* = 5 nm) and for longitudinal light polarization

Optical properties of bowtie structures after placement of single, spherical, 30-nm size nanoparticle (NP) in the midgap were also analyzed (Fig. 5). Two kinds of NP materials were considered: dielectric (SiO_2_) and conductive (Au). The ECS spectra for transversal polarization are relatively independent of the presence of nanoparticle in the midgap (results not shown) due to weak dipole-dipole coupling and will not be further discussed. For longitudinal polarization of light, the presence of NP in the midgap has a great influence on the optical properties of the bowtie structure. For the dielectric NP, the strong electric field in the midgap causes charge polarization in the nonconducting sphere. The charge accumulation process is strongly limited by the small real part of the dielectric constant of SiO_2_ material (Fig. 1d). For this reason, only a small enhancement of the electric field near the triangular tips with respect to that for a bowtie without NP is observed (Fig. 5a). The coupling dipole-dipole resonance is stronger due to electric field enhancement in the bowtie gap (Fig. 5a) which results in the resonance at a little lower energy, as shown in Fig. 5b. This electric field enhancement is the result of the dielectric screening effect and has been reported in paper [5]. Using the circuit model of a nano-optical system [36], these phenomena can be explained as an addition of a capacitor in the series L-C resonant circuit, which leads to a decrease in the resonance frequency. Enormous electric field enhancement always appears in the gap of the nanoparticle dimer or is localized at a sharp corner. Electron plasma oscillations on the surface of Au NP cause very strong enhancement of the electric field in the midgap near the triangular tips, as presented in Fig. 5a. This electric field enhancement results in stronger dipole-dipole coupling between the triangles, which causes a relatively sharp redshift of the resonance peak when compared to that for the bowtie without and with dielectric NP (Fig. 5b).

**Fig. 6:**
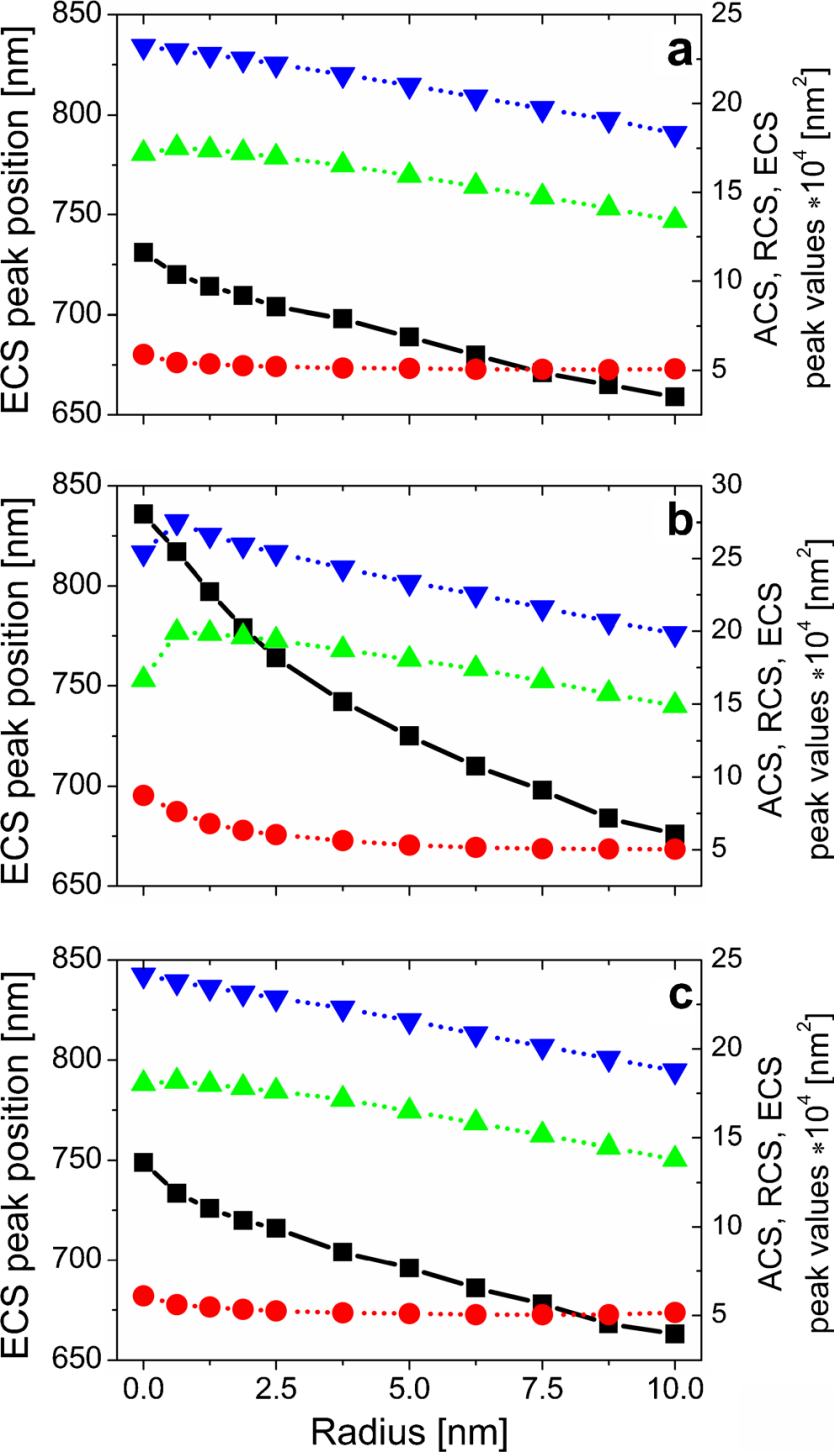
ECS peak position and ACS, RCS, and ECS peak values versus edge rounding of a bowtie without (**a**) with Au (**b**) and with SiO_2_ (**c**) nanoparticle for longitudinal incident light polarization (*black line* and *symbol*: ECS peak position, whereas *red*, *green*, *blue* correspond respectively to ACS, RCS, and ECS peak values). Color figure online

Figure 6a–c shows the dependencies of the ECS main peak position as well as the ACS, RCS, and ECS peak values on the radius of edge curvature for longitudinal polarization. When there is no NP in the bowtie gap, the difference in the peak position (Δλ = 71 nm) is noticeable for longitudinal polarization (Fig. 6a); however, the ECS peak position versus *R* still remains nonlinear. By introducing Au NP in the gap, a tunable plasmonic system can be achieved, and because of the Au presence, the tunable range can be much expanded as shown in Fig. 6b. For the arrangement with Au in the middle of nanoprism dimers, the ECS peak position Δλ was increasing significantly and reached 160 nm for longitudinal polarization (Fig. 6b). This dependence can be approximated by an exponential curve. When Au NP is replaced by a nonconductive NP such as SiO_2_, the ECS peak is slightly shifted with increasing curvature radius and Δλ equals 86 nm for longitudinal polarization (Fig. 6c). For transversal polarization, for which the weak coupling strength is observed, Δλ is equal to 56 nm and does not depend on the NP presence.

As follows from Fig. 6b, the RCS peak value for a bowtie with Au NP in the midgap and *R* = 0 is lower compared to the case when *R* ∈ < 0.625, 2.5 > nm. In contrast to the other results (see Fig. 6a, c), the dipole and strong quadrupole resonance peaks for a bowtie with sharp edges are observed (not shown). Thus, a decrease in the RCS main peak for *R* = 0 when compared to that for *R* ∈ < 0.625, 2.5 > nm (Fig. 6b) can be explained by the interaction of the dipole and quadrupole mode (Fano resonance). For the other structures analyzed, for which the results are shown in Fig. 6a, c, the above-mentioned dependencies are also valid, but because of a smaller contribution of the quadrupole mode, they are not distinctly observable.

The presented results, based on numerical simulations, could be helpful for designing and realizations of refined structure with required optical properties for selected applications. Our and previous results pointed out that it is not possible to fabricate nanoprisms with perfectly sharpened edges [13, 19], and what is more, the effect of edge rounding could be important in applications in which the far-field properties are crucial.

## Conclusions

To conclude, the numerical studies have demonstrated how geometrical modification of single and dimer nanoprisms (mainly based on rounding of their edges) influence the far-field and near-field properties. A nanoparticle made from different materials and placed in the midgap was also considered. We found that the presence of the nanoparticle there affects optical property response changes. The results presented indicate that the radius of edge curvature strongly influences the position of the plasmon peak and that this point cannot be neglected on designing plasmonic devices. Moreover, the inclusion of a nanoparticle in the gap leads to an increase in the maximum value of electric field near the nanostructure, and this effect is more pronounced for a metal than for a dielectric nanoparticle and is also manifested as a larger ECS peak shift as a function of the radius of curvature for the longitudinal polarization. These two effects are not observed for transversal polarization of incident light.

## References

[1] Larmour IA, Graham D (2011). Surface enhanced optical spectroscopies for bioanalysis. Analyst.

[2] Kneipp K, Kneipp H, Itzkan I, Dasari RR, Feld MS (2002). Surface-enhanced Raman scattering and biophysics. J Phys Condens Matter.

[3] Castro JCA, Beltrán ASC (2011). Surface plasmon resonance of a few particles linear arrays. J Electromagnet Anal Appl.

[4] Guedje FK, Giloan M, Potara M, Hounkonnou MN, Astilean S (2012). Optical properties of single silver triangular nanoprism. Phys Scripta.

[5] Ye J, van Dorpe P (2012). Plasmonic behaviors of gold dimers perturbed by a single nanoparticle in the gap. Nanoscale.

[6] Rang M, Jones AC, Zhou F, Li Z-Y, Wiley BJ, Xia Y, Raschke MB (2008). Optical near-field mapping of plasmonic nanoprisms. Nano Lett.

[7] Zhang Z, Zhang Z, Xiong Z (2010). Optical properties of silver hollow triangular nanoprisms. Plasmonics.

[8] Hermoso W, Alves TV, Oliveira CS, Moriya EG, Ornellas FR, Camargo PHC (2013). Triangular metal nanoprisms of Ag, Au, and Cu: modeling the influence of size, composition, and excitation wavelength on the optical properties. Chem Phys.

[9] Liu M, Tang ML, Hentschel M, Giessen H, Alivisatos AP (2011). Nanoantenna-enhanced hydrogen gas sensing in a single tailored nanofocus. Nat Mater.

[10] Urban AS, Lutich AA, Stefani FD, Feldmann J (2010). Laser printing single gold nanoparticles. Nano Lett.

[11] van Dorp WF, van Someren B, Hagen CW, Kruit P, Crozier PA (2005). Approaching the resolution limit of nanometer-scale electron beam-induced deposition. Nano Lett.

[12] van Kouwen L, Botman A, Hagen CW (2009). Focused electron-beam-induced deposition of 3 nm dots in a scanning electron microscope. Nano Lett.

[13] Yamaguchi G, Inoue T, Fujii M, Ogawa T, Matsuzaki Y, Okamota T, Haraguchi M, Fukui M (2008). Characteristics of light intensity enhancement of a silver nanoprism with rounded corners. J Microsc.

[14] Zhu S, Zhou W, Park GH, Li E (2010). Numerical design methods of nanostructure array for nanobiosensing. Plasmonics.

[15] Dodson S, Haggui M, Bachelot R, Plain J, Li S, Xiong Q (2013). Optimizing electromagnetic hotspots in plasmonic bowtie nanoantennae. J Phys Chem Lett.

[16] Sherry LJ, Jin R, Chad CA, Schatz GC, Duyne R (2006). Localized surface plasmon resonance spectroscopy of single silver triangular. Nano Lett.

[17] Qian J, Liu C, Wang W, Chen J, Li Y, Xu J, Sun Q (2013). Effect of edge rounding on the extinction properties of hollow metal nanoparticles. Plasmonics.

[18] McMahon JM, Wang Y, Sherry LJ, Duyne RP, Marks LD, Gray SK, Schatz GC (2009). Correlating the structure, optical spectra, and electrodynamics of single silver nanocubes. J Phys Chem C.

[19] Raziman TV, Martin OJF (2013). Polarisation charges and scattering behavior of realistically rounded plasmonics nanostructures. Opt Express.

[20] Goldys EM, Calander N, Drozdowicz-Tomsia K (2011). Extreme sensitivity of the optical properties of metal nanostructures to minor variations in geometry is due to highly localized electromagnetic field modes. J Phys Chem C.

[21] Weiland T (1977). A discretization method for the solution of Maxwell’s equations for six-component fields. Electron Commun.

[22] Clemens M, Weiland T (2001). Discrete electromagnetism with the Finite Integration Technique. Prog Electromagn Res.

[23] Dyck NC, Denomme RC, Nieva PM (2011). Effective medium properties of arbitrary nanoparticle shapes in a localized surface plasmon resonance sensing layer. J Phys Chem C.

[24] Weiland T, Timm M, Munteanu I (2008). A practical guide to 3-D simulations. IEEE Microw Mag.

[25] Palik ED (1998) Handbook of Optical Constants of Solids. Academic, San Diego, CA

[26] Bohren CF, Huffman DR (1998). Absorption and scattering of light by small particles.

[27] Novotny L (2007). Effective wavelength scaling for optical antennas. Phys Rev Lett.

[28] Myroshnychenko V, Rodríguez-Fernández J, Pastoriza-Santos I, Funston AM, Novo C, Mulvaney P, Liz-Marzán LM, García de Abajo FJ (2008). Modelling the optical response of gold nanoparticles. Chem Soc Rev.

[29] Shuford KL, Ratner MA, Schatz GC (2005). Multipolar excitation in triangular nanoprisms. J Chem Phys.

[30] Rycenga M (2009). Surface-enhanced Raman scattering: comparison of three different molecules on single-crystal nanocubes and nanospheres of silver. J Phys Chem A.

[31] Camargo P, Au L, Rycenga M, Li W, Xia W (2010). Measuring the SERS enhancement factors of dimers with different structures constructed from silver nanocubes. Chem Phys Lett.

[32] Zhou F, Li Z-Y, Liu Y (2008). Quantitative analysis of dipole and quadrupole excitation in the surface plasmon resonance of metal nanoparticles. J Phys Chem C.

[33] Luk’yanchuk B, Zheludev NI, Maier SA, Halas NJ, Nordlander P, Giessen H, Chong CT (2010). The Fano resonance in plasmonic nanostructures and metamaterials. Nat Mater.

[34] Rechberger W, Hohenau A, Leitner A, Krenn JR, Lamprecht B, Aussenegg FR (2003). Optical properties of two interacting gold nanoparticles. Opt Commun.

[35] Nordlaner P, Oubre C, Prodan E, Li K, Stockman MI (2004). Plasmon hybridization in nanoparticle dimers. Nano Lett.

[36] Engheta N, Salandrino A, Alu A (2005). Circuit elements at optical frequencies: nanoinductors, nanocapacitors, and nanoresistors. Phys Rev Lett.

